# Best achievable results need territorial familiarity: Impact of living donor liver transplant experience on outcomes after pancreaticodoudenectomy

**DOI:** 10.1016/j.amsu.2020.05.024

**Published:** 2020-05-30

**Authors:** Abu Bakar H. Bhatti, Roshni Z. Jafri, Nasir A. Khan

**Affiliations:** a Department of Hepato-Pancreatico-Biliary Surgery and Liver Transplantation, Shifa International Hospital Islamabad, Pakistan; b Department of Anesthesiology, Shifa International Hospital Islamabad, Pakistan

**Keywords:** Pancreaticodoudenectomy, Pancreatic fistula, Failure to rescue, Living donor liver transplant

## Abstract

**Background:**

Recently, benchmarks for pancreatic surgery have been proposed. Living donor liver transplantation (LDLT) is thought to have a positive impact on PD outcomes. The objective of the current study was to determine if the proposed benchmark cutoffs are achievable in an LDLT program with low to medium volumes for PD.

**Methods:**

We retrospectively reviewed patients who underwent PD between 2011 and 2018 (N = 116). Their outcomes were assessed and compared with benchmark cutoffs for pancreatic surgery based on results from high volume centers (HVC) for PD. During the same period, 759 LDLTs were performed in our center. Outcomes were further compared based on whether PD was performed in low volume (≤76/year) (Group 1) or high volume (>76/year) (Group 2) transplant years.

**Results:**

Out off 20 benchmarks, 15 (75%) were met while 19/20 (95%) were within range reported from HVC-PD. Benchmarks remained within range for biochemical leak (15.5% vs 13%, 1.3–22.7%), grade 4 complications (12.1% vs 5%, (0–14%), hospital mortality (3.8% vs 1.6%, 0–4%) and failure to rescue (24.4% vs 9%, 0–25%). There was a significant reduction in blood transfusion rate (69% vs 39.5%, P = 0.003) in group 2 while patients with at least one complication (45.5% vs 66.7%) (P = 0.04), median hospital stay (9 vs 11, P = 0.004), and median comprehensive complication index (CCI) (0 vs 20.9, P = 0.005) increased.

**Conclusion:**

Best achievable results for PD can be reproduced in LDLT programs with low to moderate PD volumes. Transition to a high volume transplant center does not confer additional improvement in outcomes.

## Highlights

∙ This is the first study to objectively assess impact of living donor liver transplant (LDLT) experience on pancreaticdoudenectomy (PD) outcomes.
∙Even with low to moderate PD volumes, LDLT programs can achieve outcomes comparbale to high volume centers.
∙Transition from low to high annual LDLT volume does not confer any additional improvement in PD outcomes.

## 1 Introduction

Traditionally, pancreaticodoudenectomy (PD) has been associated with high morbidity and mortality [1]. Over the last twenty years, marked improvement in outcomes has been observed. An obvious reduction in hospital mortality, now ranging between 1 and 5% has been reported from experienced centers [[2], [3], [4]]. High volume centers (HVC) tend to have better outcomes, supporting the notion of centralization for complex procedures [5]. Even low volume surgeons (LVS) in HVCs may have comparable outcomes due to strong support systems and ability to rescue patients from major complications [6]. Nevertheless, best outcomes are achieved in patients with minimal complications and smooth recovery which is consequent upon safe execution of surgery.

One of the major problems with outcome comparison is non-standardized reporting of outcomes, heterogeneity in patient population and absence of benchmarks for complex surgery [7]. Living donor liver transplantation (LDLT) is one of the most demanding abdominal surgical operations. Not surprisingly, many liver transplant surgeons worldwide perform complex pancreatic resections without achieving high annual volumes, due to busy transplant practice and referral trends. It is believed that skills acquired in LDLT positively impact PD outcomes [8]. Indeed, LVS for PD with a high operative mix of hepatic, biliary and gastric procedures demonstrate better outcomes than surgeons performing PD with a lower operative mix [9].

Our center is a high volume LDLT center but remains low to medium volume for PDs (<20 resections/year). A positive impact of LDLT on PD outcomes has been suggested but remains to be objectively investigated [7]. With the recently proposed benchmarks in pancreatic surgery, it is now possible to perform meaningful comparisons, with more objective data, and authenticate various speculations regarding complex surgeries [10].

The objective of the current study was to determine if the proposed benchmark cutoffs are achievable in an LDLT program with low to medium volumes for PD.

## 2 Methods

### 2.1 Treatment protocol

This was a review of patients who underwent pancreaticodoudenectomy (PD) between January 2011 and December 2018. A total of 116 patients with a diagnosis of cancer/dysplasia on final histopathology were included. Details of preoperative workup, surgical procedure and post operative follow up have been reported elsewhere [11,12]. All patients were discussed in multi disciplinary team meeting and a treatment plan was formalized. Preoperative biliary drainage was performed in patients with a total bilirubin ≥10 mg/dl or suspicion of cholangitis. Endoscopic retrograde cholangiopancreatography (ERCP) was the preferred intervention, and in unsuccessful cases percutaneous transhepatic cholangiography (PTC) was used. For the purpose of this study, we used the 8th edition of TNM classification for staging [13]. Para aortic lymphadenectomy and peri portal lymphadenectomy was routinely performed.

Nasojejunal (NJ) feeding was initiated on 2nd postoperative day. NJ tube was removed on day 4 if there was no clinical suspicion of pancreatic fistula and patient had a smooth postoperative course.

Carbepenems were administered for 5 days as routine. Patients were kept in intensive care unit (ICU) for 1–2 days. Patients were seen at 2 weeks, 3 months, 6 months and 12 months after discharge, and then annually.

### 2.2 Outcome definitions

**Fig. 1:**
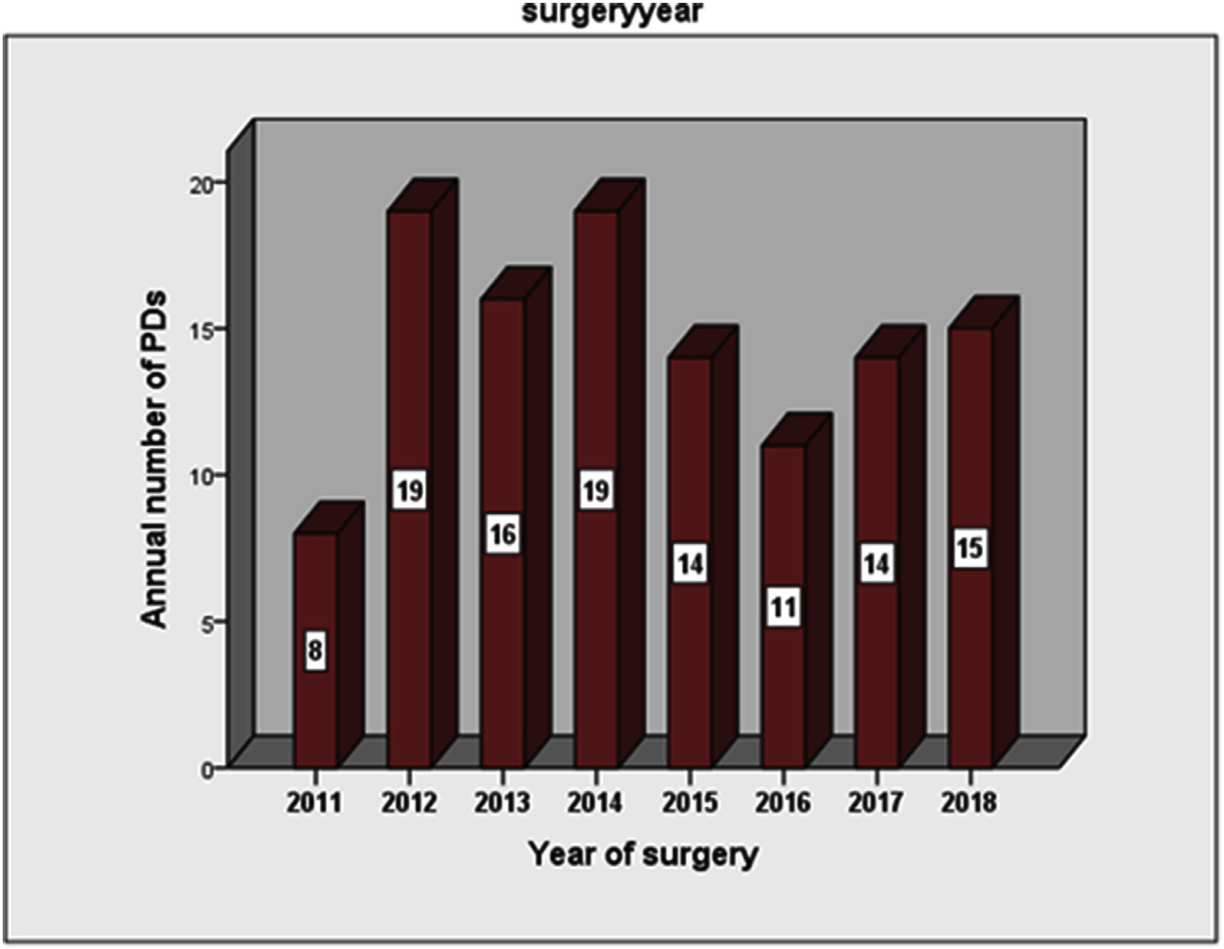
Annual number of pancreaticodoudenectomies (PDs) for malignancy.

For outcome comparison, we used the recently proposed benchmark cutoffs after PD [10]. It includes 20 intraoperative and postoperative variables with cutoffs based on results from 23 high volume centers (HVC) worldwide performing ≥ 50 pancreatic resections annually. For classification of pancreatic fistula, we used the International Study Group on Pancreatic Fistula (ISGPF) 2016 guidelines [14]. Based on the systematic review by Hata and colleagues, we classified our center as low to medium volume center (8–19 resections/year) for PD (Fig. 1) [5]. Comprehensive complication index (CCI) was calculated with CCI calculator (https://www.assessurgery.com). Failure to rescue was defined as number of deaths due to > grade 2 complications divided by total number of > grade 2 complications [15]. Patients who had multivisceral resection (MVR) were excluded from outcome analysis for blood transfusion rate, morbidity and mortality (n = 13). Patients who had MVR and <1 year follow up were excluded from analysis for comprehensive complication index (CCI) and failure to rescue (FTR) (n = 22). Actual disease free survival (DFS) and actuarial DFS was not assessed for patients with a minimum follow up of <1 year (n = 9).

Readmission rates could not be retrieved for patients operated between 2011 and 2014 and were only documented for patients operated between 2015-18 (n = 54). We further assessed the impact of low volume versus high volume LDLT years on PD outcomes. It's been shown that centers that perform >76 liver transplants annually are high volume for liver transplantation [16]. We divided our patients into two groups; group 1 had PD performed in low volume liver transplant years (2011–2014) while group 2 underwent PD during high volume transplant years (2015–2018). A total of 759 LDLTs were performed over 8 years. Out of these, 136 were performed in low volume transplant years while 623 were performed in high volume years. The annual number of transplants in group 1 was ≤70 while it was >120 in group 2.

### 2.3 Statistical analysis

We used Pearson chi-square test and Fischer exact test to determine significant differences between categorical variables. For numerical variables, Student *t*-test and Mann-Whitney *U* test were used as appropriate. Overall survival was calculated by subtracting date of death or last follow up from date of surgery. All patients with documented evidence of mortality or a loss to follow up were considered dead. All complications were recorded based on Clavien-Dindo grading [17]. Survival was estimated using Kaplan Meier survival curves and Log rank test was used to determine significance between variables. A P value < 0.05 was considered statistically significant. All analysis was performed on SPSS Statistical software package (SPSS, version 20, IBM, Armonk, NY). The unique identification number of the research was researchregistry5305 and the study was approved by the hospital ethics committee. The work has been reported in line with the STROCSS criteria [18].

## 3 Results

### 3.1 Patient characteristics

Mean age was 59 ± 12.2 (26–85) years. Male to female ratio was (80/36) 2.2:1. Median follow up was 14.7 (0.2–82) months. Preoperative biliary drainage was performed in 64 (55.1%) patients. Two patients had a biliary bypass elsewhere before they were referred for assessment of resectability. Standard PD was performed in 81 (69.8%) patients while MVR along with PD was performed in 13 (11.3%) patients as shown in Table 1. The most common underlying pathology was ampullary 59 (50.9%) adenocarcinoma.

**Table 1:** Patient characteristics and treatments received.

		Number N = 116	Percent
**Gender**	Male	80	68.9
**Pre operative drainage**	Performed	64	55.1
	Endoscopic retrograde cholangiopancreatography (ERCP)	55	47.4
	Percutaneous transhepatic cholangiography (PTC)	5	4.3
	Surgical bypass	2	1.7
	ERCP + PTC	2	1.7
**Surgical procedure**	Standard Pancreaticodoudenectomy (PD)	81	69.8
	Pylorus preserving PD	22	19
	PD + organ resection	13	11.3
**Vascular resection**	Performed	14	12
**Adjuvant treatment**	Given	72	62

Most patients had advanced (T3/T4) tumors (78/116)(67.2%) and positive nodes (82/116)(70.6%) on final histopathology (Table 2). Out of total, 104 (89.6%) patients had well/moderately differentiated tumors.

**Table 2:** Histopathological variables in patients who underwent pancreaticodoudenectomy.

		Number N = 116	Percent
**Origin**	Pancreatic	38	32.7
	Ampullary	59	50.9
	Duodenal	5	4.3
	Cholangiocarcinoma	13	11.2
	High grade dysplasia	1	0.9
**Tumor size**	T1/T2	37	31.8
	T3/T4	78	67.2
**Nodal involvement**	N0	34	29.3
	N1	47	40.5
	N2	35	30.2
**Histology (n** = **115)**	Well	7	6.1
	Moderate	97	84.3
	Poor	11	9.6
**Margins**	Positive	24	20.7
	Uncinate	21	18.1
	Hepatic margins	2	1.7
	Gastric margin	1	0.9
**Perineural invasion**	Positive	40	34.5
**Lymphovascular invasion**	Positive	45	38.8

### 3.2 Comparison with benchmark cutoffs

Out of 20 predefined benchmark cutoffs for postoperative outcomes, 15 (75%) were successfully achieved in the current study and 19/20 (95%) were within the range reported from HVCs as shown in Table 3.

**Table 3:** Comparison of benchmark cutoffs and outcomes in the current study.

	Benchmark cutoffs (range) in high volume centers	Outcomes in current study N = 116	
		Median	Range
Operative time (hours)	≤7.5 (3.4–8.6)	7.5	4–12
Median hospital stay (days)	≤15 (6–31)	10	6–70
Lymph nodes retrieved	≥16 (14–43)	29	6–82
CCI (n = −94)	≤20.9 (0–35.4)	20.9	0–100
		**Number**	**Percent**
6 month morbidity (n = 103)			
At least 1 complication	73% (43.5–89.6%)	57	55.3%
Grade 1–2	62% (30.6–86.5)	21	20.4%
Grade 3	30% (4.4–52.3)	28	27.2%
Grade 4	5% (0–14)	8	7.8%
Blood transfusions (n = 103)	≤23% (2–36.4)	57	55.3%
PF rate (Grade B/C)	≤19% (0–35.4%)	10	8.6%
Biochemical leak	≤13% (1.3–22.7)	18	15.5%
Grade B pancreatic fistula	≤15% (0–35.4)	6	5.2%
Grade C pancreatic fistula	≤5% (0–12)	4	3.4%
Severe post op bleeding	≤7% (0–14)	2	1.7%
In hospital mortality (n = 103)	≤1.6% (0–4)	4	3.8%
Failure to rescue (FTR) (n = 94)	9% (0–25)	8/32	25%
Re admission rate (n = 54)	≤21% (1.6–29.1)	6	11.1%
Microscopic positive margin (R1) rate	≤39% (2.3–67%)	24	20.7%
1 year actual DFS (N = 107)	≥53% (22.6–100%)	86	80.3%
3 year actuarial DFS (N = 107)	≥9% (0–15.4%)	–	53%

Need for intraoperative blood transfusion was the only variable that remained out of range when compared with results from HVCs i.e. 57 (55.3%) against proposed cutoff ≤ 23%, (range = 2–36.4%). The actual 1 year DFS was 80.3% against a benchmark cutoff of 53% (22.6–100%) in HVCs. Marked improvement in outcomes for 4/20 (20%) benchmark cutoffs was noted. The margin positive rate (20.7% vs 39%), grade B/C pancreatic fistula rate (8.6% vs 19%), re admission rate (11.1% vs 21%), and severe postoperative bleeding (1.7% vs 7%) were ≥ 40% lower in our center.

### 3.3 Outcomes in low vs high volume years

Table 4 demonstrates the outcome comparison between low and high volume transplant years with reference to the benchmark cutoffs. We noted a higher percentage of older (Age >70) patients in group 2 i.e. 12/62 (19.3%) vs 18/54 (33.4%), P = 0.08). There was no significant difference in 15/19 (78.9%) variables between the two groups. There was a significant increase in median CCI in group 2 (0 vs 20.9)(P = 0.005) and median hospital stay (9 vs 11 days, P = 0.004). Number of patients with at least one complication also increased (45.5% vs 66.7%) (P = 0.04). Although not significant, FTR rates also increased in group 2 (7.6% vs 36.8%, P = 0.1). Number of patients needing blood transfusion decreased (69% vs 39.5%, P = 0.003) in group 2. A trend towards reduction in positive margins was noted for group 2 (27.4% vs 12.9%, P = 0.05).

**Table 4:** Comparison of demographic, operative and clinical variables between low volume and high volume liver transplant years.

	Benchmark Cut offs and range in high volume centers	Low volume transplant years N = 62		High volume transplant years N = 54		P Value
		Median	Range	Median	Range	
**Operative time (hours)**	≤7.5	7.5	4.5–12	7	4–11	0.08
**Median hospital stay (days)**	≤15 (6–31)	9	6–21	11	7–70	0.004
**Lymph nodes retrieved**	≥16 (14–43)	30.5	6–82	28.5	11–66	0.9
**CCI (n** = **94)**	≤20.9 (0–35.4)	0	0–100	20.9	0–100	0.005
		**Number**	**Percent**	**Number**	**Percent**	
**6 month morbidity (n** = **103)**						
**At least 1 complication**	73% (43.5–89.6%)	25/55	45.5	32/48	66.7	0.04
**Grade 1–2**	62% (30.6–86.5)	12/55	21.8	9/48	18.7	0.09
**Grade 3**	30% (4.4–52.3)	12	21.8	16	33.3	
**Grade 4**	5% (0–14)	1	1.8	7	14.5	
**Blood transfusions (n** = **103)**	≤23% (2–36.4)	38/55	69	19/48	39.5	0.003
**PF rate (Grade B/C)**	≤19%	5	8	5	9.2	1
**Biochemical leak**	≤13%	10	16.1	8	14.8	1
**Grade B pancreatic fistula**	≤15	3	4.8	3	5.6	1
**Grade C pancreatic fistula**	≤5	2	3.2	2	3.7	1
**Severe post op bleeding**	≤7% (0–14)	1	1.6	1	1.8	1
**In hospital mortality N** = **103**	≤1.6% (0–4)	1/55	1.8	3/48	6.2	0.3
**FTR (n** = **94)**	9% (0–25)	1/13	7.6	7/19	36.8	0.1
**R1 rate**	≤39% (2.3–67%)	17	14.6	7	6	0.05
**1 year actual DFS (N** = **107)**	≥53% (22.6–100%)	49/62	79	37/45	82.3	0.6
**3 year actuarial DFS (N** = **107)**	≥9% (0–15.4%)	–	40.2	–	71	0.08

## 4 Discussion

The current study demonstrates comparable outcomes after PD from an LDLT program despite modest annual PD volume. Although it has been suggested that skills acquired in liver transplantation can impact PD outcomes, this is the first study to objectively investigate this assumption, based on recently proposed benchmarks from high volume centers worldwide [10].

There remains a debate as to what constitutes high volume for PD [[19], [20], [21]]. Variable cutoffs have been used but a recent systematic review demonstrated benefit in terms of postoperative outcomes in centers performing ≥ 21 PDs annually [5]. We have compared our postoperative outcomes with HVCs for PD (≥50 resections/year) based on 20 proposed variables and the results were comparable for most benchmarks [10]. In the current study, 19/20 (95%) outcome variables were comparable despite low to moderate PD activity in our center.

Pancreatic fistula remains the most challenging and devastating complication after PD. Certain factors like pancreatic texture, duct diameter, and body mass index (BMI) have been implicated as risk factors for pancreatic fistula formation [[22], [23], [24], [25]]. We observed a markedly reduced rate of clinically relevant PF (type B/C) when compared with the PF rates from HVCs. A similar difference was noted for margin positivity, nodal yield, postoperative bleeding and re admission rates. The high nodal yield can be attributed to the inclusion of para aortic lymphadenectomy as a standard procedure along with PD. We believe that the comparable results were achieved due to territorial familiarity (TF) with PD. TF emphasizes upon acquisition of knowledge and experience that leads to *increased anatomical familiarity* and skill set to perform procedures of *high technical complexity*. TF for a complex surgical procedure like PD can be achieved, by performing the same procedure repeatedly for varied clinical presentations and stages of the disease, or frequent exposure to other technically complex procedures in the same anatomical territory.

Despite lack of high volume exposure to PD, transplant surgeons might have TF for PD due to factors described in Table 5. All operating surgeons had outstanding exposure to hepato-pancreatobiliary and transplant procedures. Our intensive care unit team has a vast experience in managing liver and kidney transplant patients, adult and pediatric cardiac interventions, oncological and neurosurgical procedures. A preoperative biliary drainage procedure was performed in >50% patients in the current study. Although we favor upfront surgery in patients with total bilirubin <10 mg/dl, many patients are referred from other hospitals and have already undergone endoscopic stenting.

**Table 5:** Factors leading to anatomical familiarity and attainment of technical complexity with potential advantages during pancreaticodoudenectomy.

**Anatomical familiarity**	Surgical maneuver	Potential advantage during pancreaticodoudenectomy (PD)
Hepatic artery	Isolation of hepatic arterial system including right, left, proper hepatic artery and occasionally common hepatic artery for arterial anastomoses	Exposure to superior border of pancreas
		Identification and ligation of gastrodoudenal artery
		Excision of common hepatic artery lymph node
Portal vein	Isolation of portal vein and its branches Temporary portocaval shunts	Portal venous resection and reconstruction in borderline tumors
	Portal vein anastomosis	
Superior mesenteric vein (SMV)	Use of SMV jump grafts in patients with portal vein thrombosis	Exposure to inferior border of pancreas
		SMV resection and anastomoses in borderline tumors
Hilum	High hilar dissection during recipient and donor hepatectomy	Hilar lymphadenectomy
		Multi visceral resections
**Technical Complexity**		
Portal hypertension Coagulopathy Spontaneous bacterial peritonitis (SBP)	Dissection of porta hepatis in the presence of pericholedochal varices, friable or frozen tissues due to previous surgery or SBPs	PD in cases with previous surgical attempts PD with concomitant pancreatitis Distorted anatomy due to previous ERCP/PTC Locally advanced distal cholangiocarcinoma PD in well compensated cirrhotics
Living donor surgery	Ensuring safe graft procurement and preserving integrity of the future liver remnant mandating careful preoperative planning	Better understanding of hepatic venous and biliary anatomy
		Effective in complex resections like central and extended hepatectomies along with PD
Complex anastomoses	Complex arterial and biliary anastomoses under loupe magnification	Arterial resection and reconstruction in PD
		Challenging pancreaticojejunal anastomoses High hepaticojejunostomies in cholangiocarcinoma
Portal flow modulation	Portal flow modulation by portocaval shunt/splenic artery ligation/splenectomy for portal hypertension	Handy when performing total pancreatectomy or splenectomy

We noted a high but within range of failure to rescue (FTR) rate in the current study. FTR has become more relevant as a quality of care indicator and a high FTR rate is suggestive of deficiencies in ancillary care services [15]. In addition, the tremendous cost implications in rescuing patients from major complications play a decisive role in high FTR rates [26]. In our context, the relatively high rate of FTR is partially attributable to non popularity of health insurance systems, most patients pay out of pocket and suffer tremendous financial burden incase of prolonged hospitalization due to a serious complication [27,28].

We separately assessed outcomes in low volume and high volume transplant years. Blood transfusion rate dropped significantly in the later period. The reduction in rate of intraoperative transfusion corresponds to increasing evidence of deleterious effects of transfusions on outcomes after abdominal surgery [29,30]. There was a significant increase in hospital stay, median CCI and number of patients who experienced a morbidity. We believe that advancing patient age undergoing PD, unanticipated increase in transplant volume with resource restriction, and initiation of fellowship training program in hepatobiliary surgery and liver transplantation in the later period, might have partially contributed to these findings [[31], [32], [33]]. Nevertheless, benchmarks were still achieved for these variables. This suggests that even low volume LDLT exposure allows attainment of TF with PD. This is particularly important for centers performing relatively lower numbers of LDLTs annually.

In the current study, 20 benchmark variables with their cutoffs based on results from some of the best centers for PD were compared with an LDLT program with modest annual PD experience. LDLT imparts TF for PD and leads to outcomes comparable to experienced centers worldwide. Since most benchmark cutoffs were achieved in low and high volume transplant years, transition from a low to high volume transplant center does not necessarily result in further improvement in PD outcomes. The minimum LDLT experience that develops TF for PD remains to be determined.

## Ethical approval

The hospital ethics committee at Shifa International Hospital, approved the study. The reference number was IRB # 238-728-2019.

## Sources of funding

No funding was received.

## Author contribution

AHB: Contributed to concept, design, data collection, analysis, writing and critical review of the manuscript.

RZJ: Contributed to data collection, analysis, writing and critical review of the manuscript.

NAK: Contributed to concept, design, writing and review of the manuscript.

## Research registration Unique Identifying Number (UIN)

1 Name of the registry: Research Registry.

2 Unique Identifying number or registration ID: 5305.

3 Hyperlink to the registration (must be publicly accessible): https://www.researchregistry.com/browse-the-registry#home/registrationdetails/5e0f25267c018a00167dd34e/

## Guarantor

Abu Bakar Hafeez Bhatti.

## Declaration of competing interest

None of the authors have any potential conflicts of interests.
